# A Neonatal Patient Diagnosed with a *COL4A1* Mutation Presenting with Hemorrhagic Infarction and Severe Jaundice

**DOI:** 10.1155/2022/1594364

**Published:** 2022-10-14

**Authors:** Akihiro Kirimura, Hajime Yasuhara, Soshi Hachisuka, Kumiko Takagi, Reiko Ebisu, Ayako Ohgitani, Hideki Minowa

**Affiliations:** Department of Neonatal Intensive Care Unit, Nara Prefecture General Medical Center, Nara, Japan

## Abstract

We report a patient diagnosed with a *COL4A1* mutation in the early postnatal period. Patients with early postnatal jaundice, intracranial lesions that are negative for TORCH syndrome, and recurrent hemolytic anemia should be suspected of having a *COL4A1/COL4A2* gene mutation.

## 1. Introduction

*COL4A1* and *COL4A2* are genes encoding molecular chains that are major components of type IV collagen, which is found in basement membranes throughout the human body. Mutations in the *COL4A1* and *COL4A2* genes are known to cause schizencephaly and porencephaly and have been increasingly reported in recent years. Mutations in the *COL4A1* and *COL4A2* genes cause a wide variety of symptoms in the cerebrovascular, renal, ocular, cardiac, and muscular regions and are referred to as *COL4A1/COL4A2* mutation-related disorders [1].

We experienced a case of early jaundice requiring exchange transfusion and intracranial lesions suspicious for the TORCH syndrome, which led to an early diagnosis of a *COL4A1* mutation. We report the clinical features of the case together with a discussion of the recent literature.

## 2. Case Report

The mother was a 23-year-old woman with 2 pregnancies and 1 delivery, a blood type of O/Rh plus, and no pregnancy complications. She had a spontaneous vaginal delivery at 39 weeks and 0 days gestation. The mother tested negative for syphilis, hepatitis B, hepatitis C, human immunodeficiency virus, chlamydia, and Group B *Streptococcus*. Her rubella antibodies were less than 8-fold. We did not screen for cytomegalovirus (CMV) and toxoplasma antibodies. The patient was a female infant with a birth weight of 2450 g (−1.56 SD) and a head circumference of 28.5 cm (−3.41 SD) with Apgar scores of 8 (1-minute score) and 9 (5-minute score). Due to unstable oxygen saturation after birth, she was given oxygen at FiO2 0.3 in the incubator under observation, but on Day 1, her total bilirubin level rose to 13.2 mg/dL (225.7 *μ*mol/L), her jaundice worsened, and she was transferred to our department on the same day.

**Figure 1:**
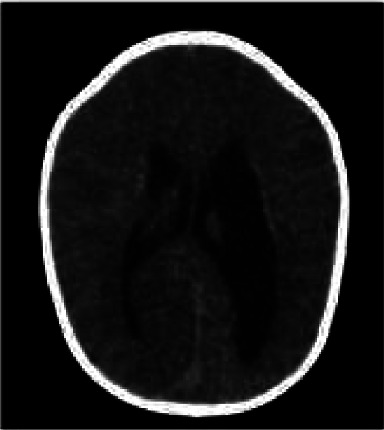
Brain CT, ventricular enlargement, and periventricular calcification.

At the time of admission, her general vitality was somewhat poor, and there were myoclonus-like involuntary movements of the limbs that were suspected to be irritating. She had a small head but did not show any disease-specific facial abnormalities. No other obvious external deformities were observed. Blood tests at the time of admission showed an elevated level of CK (4050 U/L) and hyperbilirubinemia (13.7 mg/dL [234.3 *μ*mol/L]). On admission, head ultrasonography showed marked enlargement of the left ventricle and hyperintense areas in the bilateral thalamus, and a simple brain CT was performed on the same day, which showed bilateral ventricular enlargement with left-sided predominance, periventricular calcification, and ischemic changes (Figure 1).

Initially, we suspected jaundice due to ABO blood group incompatibility. Phototherapy was started on Day 1, and intravenous immunoglobulin was administered, but the total bilirubin level rose to 15.2 mg/dL (259.9 *μ*mol/L), so an exchange transfusion was performed on the same day. After Day 2, the total bilirubin level gradually decreased, and phototherapy was completed on Day 7. Her blood type was A Rh plus, and anti-A antibodies derived from maternal blood were observed, but the diagnostic criteria for ABO blood group incompatibility were not met, as anti-A antibodies were 128-fold (<512-fold) in maternal serum and 4-fold (<8-fold) in the patient's serum.

**Figure 2:**
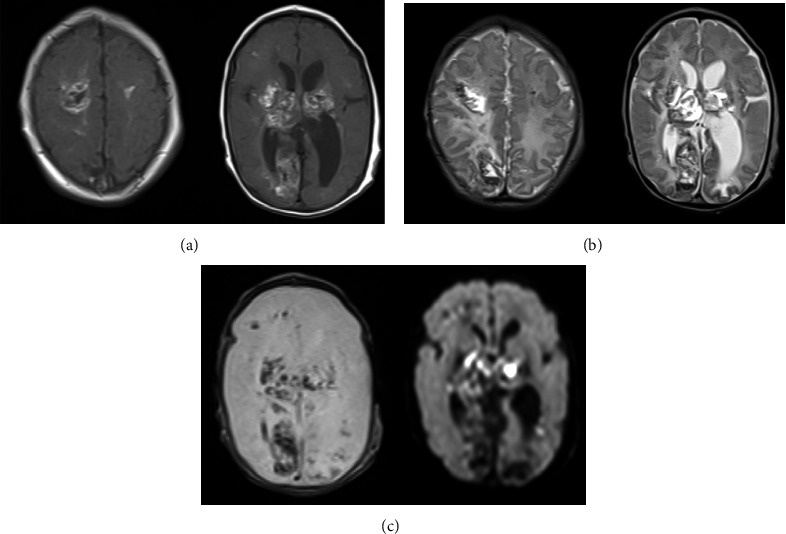
Brain MRI, multiple hemorrhagic infarcts with intraventricular perforation. (a): T1-weighted images, (b): T2-weighted images, and (c): T2^*∗*^ and DWI images.

For the intracranial lesion, the TORCH syndrome was suspected, but the patient's CMV-IgM antibody, Toxoplasma IgM antibody, and herpes simplex virus IgM tests were all negative. In addition, a real-time PCR test using the infant's urine was also negative for CMV DNA. There were no pathological abnormalities in coagulation and hemostatic function, and protein C and protein S activities were not significantly decreased. An electroencephalogram (EEG) performed on Day 3 showed reproducible sharp waves in the left hemisphere and slow wave rhythms in the bilateral occipital regions, which suggested epilepsy. A simple brain MRI was performed on Day 10 and showed multiple hemorrhagic infarcts with intraventricular perforation and cerebellar atrophy (Figure 2). Rhythmic myoclonus-like seizures appeared on Day 23, and she was started on oral phenobarbital. Simple brain MRI was performed on Day 25 and did not reveal any new lesions.

She developed anemia (Hb 9.1 g/dL) on Day 18 and was started on oral iron and subcutaneous erythropoietin. However, her Hb level decreased to 6.1 g/dL by Day 29, and red blood cell transfusion was performed by Day 30. After the transfusion, her Hb level remained in the 910 g/dL range, and her seizures were well controlled, so she was discharged on Day 39. After discharge, the anemia worsened again to a Hb level of 6.7 g/dL on Day 53, and she was hospitalized on Day 54 and underwent a second red blood cell transfusion. Since then, there has been no recurrence of anemia requiring a red blood cell transfusion.

We suspected a *COL4A1/COL4A2* mutation as the cause of the early jaundice and perinatal hemorrhagic infarction, and with the consent of her parents, we performed a genetic investigation of the patient using next generation sequencing and identified a heterozygous missense mutation in the *COL4A1* gene (p. Gly586Asp, chr13: 11018625). Although this is a novel variant that is not registered in the ClinVar database, it is a glycine substitution in the triple helix domain, which is the most frequent cause of porencephaly and schizencephaly in the previous reports and is considered to be a pathological variant. Therefore, we diagnosed the patient with *COL4A1*-related syndrome. We also examined the parents' genes and found no abnormalities in the *COL4A1* gene, indicating that the mutation was de novo.

At present, she is 10 months old. Her elevated level of CK has improved, and there has been no sign of recurrence of jaundice or anemia requiring a red blood cell transfusion, but she has persistent intractable seizures and developed hypsarrhythmia shown on EEG at 6 months of age. She was diagnosed with West syndrome and is being managed with multiple antiepileptic drugs and ACTH therapy. She is still not cervically stable and is developmentally delayed. Although COL4A1-related syndromes are known to cause cardiac, renal, and ocular abnormalities, she had no obvious complications on echocardiography, abdominal ultrasound, or ophthalmologic examination.

## 3. Discussion

In this case, the following features led to the early diagnosis of *COL4A1/COL4A2*-related syndromes: (1) intracranial lesions suggestive of the TORCH syndrome were found at birth, but all related tests were negative; (2) severe hemolytic jaundice could not be explained by ABO incompatibility; and (3) the progression of anemia requiring a blood transfusion despite standard treatment.

Gould et al. found *COL4A1* mutations in humans and mouse familial porencephaly, and validation in mice showed that mutations in the *COL4A1* gene cause vascular fragility leading to cerebral hemorrhage and porencephaly [2, 3]. *COL4A2* has a low penetrance of mutations, which makes linkage analysis difficult. However, *COL4A2* mutations have recently been confirmed to cause cerebral hemorrhage as well [4, 5]. Cerebral hemorrhage during fetal life causes damage to the neural tissue, resulting in schizencephaly or porencephaly at birth. Both schizencephaly and porencephaly can lead to a variety of neurological symptoms, such as cerebral palsy, mental retardation, and epilepsy, after birth. The mechanism causing cerebral hemorrhage in *COL4A1/COL4A2*-related syndromes has not yet been explored, but it is thought to be caused by a combination of genetic factors, such as mutations in the *α*1 chain of type IV collagen, which impair the stability of basement membranes and weaken the cerebral vascular tissue, and environmental factors, such as pressure and trauma associated with delivery [2, 3]. Tomotaki et al. reported that 15 (21%) of 61 patients with porencephaly and 10 patients with schizencephaly had *COL4A1* mutations (5 of which were de novo mutations) [6]. Meuwissen et al. performed genetic examinations of 183 patients with cerebral hemorrhage or porencephaly and their parents and identified *COL4A1* and *COL4A2* mutations in 24 patients (13%) (21 *COL4A1* mutations and 3 *COL4A2* mutations, including 10 de novo mutations) [1]. The pathogenesis of hemolytic anemia in the neonatal period has not yet been explored, but it is thought that the dysfunction of basement membranes leads to the destruction of red blood cells via the vascular system and reticuloendothelial system [7]. In most of the reported cases, anemia tends to improve within the first few months after birth. This is thought to be related to the development and maturation of the skeletal and nonskeletal components of erythrocytes. Mentzer et al. showed that free 2,3-diphosphoglycerate (2,3-DPG), present in neonatal erythrocytes, causes severe mechanical fragility in the erythrocytes of infants with hereditary erythrocytosis [8]. The decrease in free 2,3-DPG associated with the transition from fetal hemoglobin to adult hemoglobin results in a reduction in hemolytic anemia, and the same mechanism may explain the course of hemolytic anemia in *COL4A1/COL4A2*-related syndromes.

There is no fundamental treatment for *COL4A1/COL4A2*-related syndromes, and only symptomatic treatment with antiepileptic drugs and rehabilitation is available for epilepsy and psychomotor retardation caused by damage to the brain tissue. Our patient will also require extensive treatment for neurological symptoms that may develop in the future, in addition to her symptoms caused by West syndrome. On the other hand, the identification of the *COL4A1* mutation was a great advantage for us in explaining the future condition and treatment prospects to the family in detail.

## 4. Conclusion

Early postnatal jaundice, intracranial calcification, and hemorrhagic infarction led us to identify the *COL4A1* mutation. Neurological symptoms and intracranial lesions are not the only symptoms of these mutations, but jaundice and recurrent hemolytic anemia can also lead to diagnosis in the neonatal period.

It is necessary to suspect *COL4A1/COL4A2*-related syndromes in intracranial lesions that are negative for the TORCH syndrome. In this case, identification of the gene mutation was important for future prediction of the disease and genetic counseling for the family.

## Data Availability

Data sharing is not applicable to this article as no new data were created or analyzed in this study.
